# Evaluation of a multicomponent programme for the management of musculoskeletal pain and depression in primary care: a cluster-randomised clinical trial (the DROP study)

**DOI:** 10.1186/s12888-016-0772-2

**Published:** 2016-03-16

**Authors:** Enric Aragonès, Germán López-Cortacans, Antonia Caballero, Josep Ll. Piñol, Elisabet Sánchez-Rodríguez, Concepció Rambla, Catarina Tomé-Pires, Jordi Miró

**Affiliations:** Atenció Primària Camp de Tarragona; Institut Català de la Salut, Tarragona, Spain; Atenció Primària Terres de l’Ebre, Institut Català de la Salut, Tortosa, Spain; Institut Universitari d’Investigació en Atenció Primària (IDIAP) Jordi Gol, Barcelona, Spain; Chair in Pediatric Pain URV-Fundación Grünenthal and Unit for the Study and Treatment of Pain – ALGOS, Catalonia, Spain; Research Center for Behavior Assessment (CRAMC), Department of Psychology, Universitat Rovira i Virgili, Catalonia, Spain; Institut d’Investigació Sanitària Pere Virgili, Universitat Rovira i Virgili, Catalonia, Spain; Centre d’Atenció Primària de Constantí, Carrer dels Horts, 6, 43120 Tarragona, Constantí Spain

**Keywords:** Depressive disorder, Musculoskeletal pain, Primary health care, Care management, Patient education

## Abstract

**Background:**

Chronic musculoskeletal pain and depression are very common in primary care patients. Furthermore, they often appear as comorbid conditions, resulting in additive effect on adverse health outcomes. On the basis of previous studies, we hypothesise that depression and chronic musculoskeletal pain may benefit from an integrated management programme at primary care level. We expect positive effects on both physical and psychological distress of patients.

**Methods:**

*Objective:* To determine whether a new programme for an integrated approach to chronic musculoskeletal pain and depression leads to better outcomes than usual care.

*Design:* Cluster-randomised controlled trial involving two arms: a) control arm (usual care); and b) intervention arm, where patients participate in a programme for an integrated approach to the pain-depression dyad.

*Settings:* Primary care centres in the province of Tarragona, Catalonia, Spain,

*Participants:* We will recruit 330 patients aged 18–80 with moderate or severe musculoskeletal pain (Brief Pain Inventory, average pain subscale ≥5) for at least 3 months, and with criteria for major depression (DSM-IV).

*Intervention:* A multicomponent programme according to the chronic care model. The main components are care management, optimised antidepressant treatment, and a psychoeducational group action.

*Blind measurements:* The patients will be monitored through blind telephone interviews held at 0, 3, 6 and 12 months.

*Outcomes:* Severity of pain and depressive symptoms, pain and depression treatment response rates, and depression remission rates.

*Analysis:* The outcomes will be analysed on an intent-to-treat basis and the analysis units will be the individual patients. This analysis will consider the effect of the study design on any potential lack of independence between observations made within the same cluster.

*Ethics:* The protocol was approved by the Research Ethics Committee of the Jordi Gol Primary Care Research Institute (IDIAP), Barcelona, (P14/142).

**Discussion:**

This project strengthens and improves treatment approaches for a major comorbidity in primary care. The design of the intervention takes into account its applicability under typical primary care conditions, so that if the programme is found to be effective it will be feasible to apply it in a generalised manner.

**Trial registration:**

ClinicalTrials.gov: NCT02605278; Registered 28 September, 2015.

## Background

Pain and depression are common and relevant conditions in primary care patients [1, 2]. At this level of care 40 % of visits are related to pain, and musculoskeletal ailments represent the principal chronic pain disorders, particularly lower back pain and joint pain [3–5]. Major depression is present in 14 % of all primary care patients [6] and most of them (up to 85 %) also report pain-related symptoms [7, 8].

When depression and pain co-occur, both disorders can have an additive adverse effect on health and its management: the pain increases the complexity in the treatment of depression and interferes with recovery, while depression has a similar effect on the therapeutic response for the pain [9, 10].

Treatment of individuals with chronic pain and depression is complex. For example, antidepressants, which are a well-established and effective treatment for depression, have also been used for musculoskeletal pain disorders but the evidence for their usefulness when applied in this way is controversial [11]. Care management programmes for depression have shown promising results to improve the clinical outcomes in depressed patients [12], and that interventions are effective and feasible within our healthcare system [13, 14]. In turn, psychoeducational programmes and programmes supporting self-management of pain have shown to be somewhat effective for lower back and joint pains, with additional benefits on psychological distress [15]. Although of potential interest, these treatment programs are often difficult to apply in primary care, mostly because their broad format and complex execution negatively affect their feasibility in that setting [16, 17].

It therefore seems that a strategy for the joint management of chronic pain and depression is a reasonable option that could be expected to produce a synergistic effect on clinical outcomes. Based on this premise, in a study performed in primary care settings Kroenke et al. developed and tested a programme that integrated optimised antidepressant therapy (actively managed by a nurse care manager) and a psychoeducational behavioural intervention for pain self-management. There was evidence for substantial improvement in the depression symptoms as well as moderate improvements in the pain and the functional interference they were causing [18]. However, this efficacy and usefulness documented in United States health settings have not been investigated in other locations.

Our hypothesis is that both depression and chronic musculoskeletal pain will benefit from an integrated management programme for pain-depression comorbidity, established at the primary care level in our health system. Positive effects can be expected, with regard to the physical as well as psychological adjustement of patients.

## Methods/design

### Aim

The general objective is to study whether a programme to manage chronic musculoskeletal pain and depression in primary care results in better outcomes when compared to those achieved through the usual approach. The specific objectives are to determine, over the course of a 12-month monitoring period, the effectiveness of this programme on depression severity, pain severity, and therapeutic response rates for pain and depression.

### Design

**Fig. 1:**
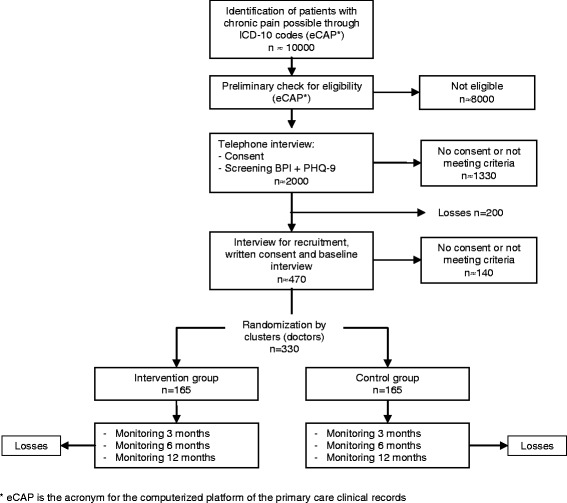
Flowchart: sampling, recruitment, randomization and monitoring of patients

A controlled trial will be conducted involving a random assignment of clusters (patients registered with the same primary care physician) to two study groups: a) control group undergoing usual care; and b) treatment group, where participants will undergo a program for the integrated management of chronic pain and depression (Fig. 1).

### Setting and study sample

The study will be conducted at the Institut Català de la Salut (Catalan Health Institute) primary care centres in the province of Tarragona, Catalonia, Spain.

Patients will be selected from the rosters of the participating physicians using the following inclusion criteria: aged 18 to 80 years old, experience moderate or severe musculoskeletal pain (average pain intensity scale on the Brief Pain Inventory (BPI) scale ≥ 5 points) for at least three months despite analgesic treatment, and have diagnostic criteria for major depressive episode (DSM-IV).

The following patients will be excluded: those suffering from any physical, psychological, or language-related limitation, or any concurrent illness that impedes their comprehension or participation in the evaluations; patients with a psychotic disorder, bipolar disorder, or alcohol or drug dependencies; and patients who are pregnant or lactating. Also considered as ineligible will be patients with an established diagnosis of fibromyalgia or somatization disorder; those with a workplace disability claim currently in progress; and those expecting an intervention for a joint prosthesis during the next 12 months. If a patient is being treated with antidepressants, this will not be a criterion for exclusion as long as the patient shows major depression criteria at the time of entering into the study.

### Random assignment to the study groups

To avoid potential contamination between the intervention and usual care, which could take place if the same physician were attending patients from the intervention group and the control group, the patients will be assigned to the study groups by clusters, with each cluster comprising patients belonging to the same physician’s roster.

The clusters from each primary care centre will be assigned to either the intervention group or control group by someone not otherwise involved in the study (Dr. J Basora, Spanish Society of Family and Community Medicine), following a simple randomised procedure using the List Randomiser tool at www.random.org. The physicians will commit to participate in the study prior to this assignment, and the patients will accept their recruitment without knowing which study group they will be assigned to.

### The multicomponent program: basic characteristics

The intervention consists of a programme that integrates and organises several components, designed according to the basic premises of the chronic care model [19] including the following main components:

#### Optimised management of major depression

Depression will be managed based on the algorithms and recommendations of the comprehensive clinical guide in the computerised primary care clinical records. This is a computerised system used to support clinical decisions that has been developed based on the most recent clinical practice guidelines for major depression [20, 21]. It assists physicians with decisions regarding diagnosis, treatment, and monitoring of major depression; systems for recording and retrieving information on a patient’s clinical status; and automated alerts for clinical situations showing poor control of the illness or risk factors [22].

#### Care management

The care manager is a psychologist who, based on the algorithms and recommendations from the computerised guideline, will support and collaborate with the treating physician in managing the patient, while also providing support for suitable compliance with the treatment by the patient and preventing discontinuities in the care. The care manager will also participate in the monitoring of the patients. Periodic follow-up will be carried out by telephone. Typically, these phone contacts will occur monthly during the first two months of the study, then every other month after that. The content of the telephone contact is structured and includes: monitoring of symptoms with the PHQ-9 (Patient Health Questionnaire) scale, the MINI scale for suicide risk (Mini International Neuropsychiatric Interview), and the GAF scale (Global Assessment of Functioning) to evaluate the impact of the depression on the patient’s functioning; as well as evaluating the patient’s compliance with treatment and identifying any difficulties in this area. These contacts will also provide the patients with therapeutic advice and reminders about upcoming visits.

On the basis of the information available through the computerised system, the care manager will provide the treating physician with information that will allow him or her to take the best decisions about health care. The care manager will also collaborate with the attending physician to ensure that these decisions (treatment, psychoeducation, referrals, new visits, etc.) are taking place effectively. Supervision and approval of this activity will be performed on a weekly basis by an expert member of the research team.

#### Psychoeducational intervention programme for patients with chronic pain and depression

This is a group-based psychoeducational programme with a cognitive-behavioural orientation. It promotes understanding and self-management of depression and pain as well as the related difficulties, in order to facilitate the acquisition of adaptive strategies for managing these conditions on a day-to-day basis and provide incentives for the patient to play an active role in their illness management [23, 24].

The programme is structured into 9 weekly sessions of 2 h, led by the care manager. These sessions include a presentation of the content and dialogue, as well as interaction between the participants. In order to promote the active and independent role of the patient, “homework” is assigned after each session, which will be reviewed at the beginning of the following session. The content of the psychoeducational sessions covers the following areas: understanding of pain; managing emotions; basic relaxation techniques; cognitive restructuring strategies; problem solving; establishment of life goals; relationships between pain and physical activity, healthy postures, and sleep; maintenance of the strategies learned; and preparation of plans to be applied in the event of temporary setbacks.

In order to facilitate the sessions, a teaching manual is made available along with other supporting materials (slide-based presentations, brochures, and forms).

### Usual care (control group)

The physicians will treat the patients assigned to the usual care group based on their own, unrestricted criteria, using any resources available that they may consider appropriate.

### Procedure for identifying and recruiting patients

The following procedure will be used to select and recruit the patients:

1. Using the list of active diagnoses in the computerised clinical records, a search will be performed for cases among the rosters of the participating physicians. The search codes and diagnoses (ICD-10; International Classification of Diseases, 10^th^ Edition) will be: M15 (polyarthrosis), M16 (coxarthrosis), M17 (gonarthrosis), M19 (other arthrosis), M47 (spondylosis), M50-M51 (disc disorders), M54.2 (cervicalgia), M54.3 to M54.5 (lumbago), M54.6 to M54.9 (dorsalgia), M25.5 (joint pain), M79.6 (limb pain), R52.1-R52.2 (chronic pain).
2. Patients identified using these diagnoses will have their clinical history reviewed manually. Based upon the information available, patients will be ruled out if it is established that they do not meet inclusion criteria.
3. The remaining individuals will be sent a letter with general information about the study and an invitation to participate. They will be notified of an upcoming telephone contact to verify their eligibility.
4. Two weeks later they will receive a telephone call during which, with the patient’s consent, screening tests will be administered in order to identify the individuals with moderate to severe pain (BPI) for at least 3 months, as well a positive screening for major depression (PHQ-9).
5. Patients positively screened for chronic pain and depression will be selected for an in-person interview with an independent interviewer to confirm whether they meet the inclusion/exclusion criteria and to obtain their informed consent in writing.

### Measurements

Standardised self-report scales will be given in person (during baseline interview) and on the telephone (follow-up interviews at 3, 6, and 12 months after recruitment). Questionnaires will be administered by an independent qualified interviewer. The interviewer will be blind to participant’s group.

### Measurement variables and tools (Table 1)

**Table 1:** Study variables

Instrument	Assessment area	Time (s) of assessment
Brief Pain Inventory (BPI). Average pain scale	Pain severity	Screening
Patient Health Questionnaire (PHQ-9)	Depression symptoms	Screening
Sociodemographic data form	Age, sex, marital status, educational level, labour status, social class	Baseline
SCID (DSM-IV). Major depression module	Major depression diagnoses	Baseline
Duke Severity of Illness Checklist (DUSOI)	Medical comorbidity	Baseline
PRIME-MD. Anxiety and dysthymia modules	Common psychiatric comorbidity	Baseline
Brief Pain Inventory (BPI).	Pain severity and interference	Baseline, 3, 6 and 12 months
Hopkins Symptom Checklist (HSCL-20)	Severity of depressive symptoms, remission and response rates	Baseline, 3, 6 and 12 months
EuroQoL-5D	Health-related quality of life	Baseline, 3, 6 and 12 months
Sheehan Disability Inventory (SDS)	Disability due to psychological problems	Baseline, 3, 6 and 12 months
Form	Days of disability leave from work or interference with usual activities in the last month	3, 6 and 12 months
Patient Global Impression of Change (PGIC)	Patient’s perspective about the efficacy of treatment	3, 6 and 12 months
Use of health resources questionnaire	Number of primary care, specialist and emergency visits, and hospitalisations due to mental health or pain problems	3, 6 and 12 months
Satisfaction with care form	Patient’s satisfaction with clinical care received for depression and pain problems	3, 6 and 12 months
Exploration of the computerised database of pharmaceutical prescription and invoicing	Pharmacological treatments for pain and depression. Medical prescription and patient consumption	3, 6 and 12 months

#### Main outcome variables

In accordance with the aims of this study, the major outcome variables are the measurements of depression and pain severity as continuous variables, pain response rate, and depression response and remission rates.

The *severity of depressive symptoms* will be measured by means of the HSCL-20 (Hopkins Symptom Checklist, 20 items) [25]. The items are rated according to the presence of the symptom in the preceding two weeks on a Likert scale with five response options, from “not at all” (0 points) to “extremely” (4 points). The overall score, which can range between 0 and 4 points, is obtained by calculating the average of the 20 items.

*Clinical remission of depression* is defined as complete relief of symptoms and return to full functioning [26] An average point score less than or equal to 0.5 is an operational indicator of depression remission [27].

*Depression response to treatment* is a defined as a 50 % reduction in the severity of the symptoms with respect to the baseline HSCL-20 score [26].

The *pain intensity* and *pain interference* will be measured using the 15-item version of the Brief Pain Inventory [28, 29]. The BPI evaluates two dimensions: the intensity of the pain and its interference with everyday activities. The intensity of the pain is measured using multiple domains (worst pain experienced, minimum pain, average pain, and current pain), all of which are to be scored using a numerical rating scale from 0 (no pain) to 10 (worst pain). Interference with functioning is also measured using numerical rating scales from 0 (no interference) to 10 (total interference).

A *therapeutic response of pain* is considered relevant when a 30 % reduction is observed with respect to the baseline score [18].

*Health-related quality of life* will be measured with the EuroQol-5-D questionnaire [30, 31]. The EQ-5D has five scales (mobility, self-care, usual activities, pain/discomfort, and anxiety/depression) with three levels of severity (no problems, some problems, and extreme problems). A global score from 1 (the best state of health) and 0 (like being dead) can be obtained. The second part records the subject’s self-assessed health on a Visual Analogue Scale from 0 (the worst health status) to 100 (the best health status).

#### Secondary variables and effect modifiers

At baseline:

- Sociodemographic information: sex, age, marital status, education, labour status, and social class based on occupation [32].
- The severity of physical comorbidity will be measured using the Duke Severity of Illness Checklist (DUSOI) [33, 34]. For each diagnosis of a physical nature, a score is assigned to the symptoms, complications, prognosis and expected response to treatment. The overall severity of the patient is evaluated from 0 to 100.
- To assess the most common psychiatric comorbidity in depressed patients we will use the dysthymia and anxiety sections of the Primary Care Evaluation of Mental Disorders (PRIME-MD). This interview can generate a range of diagnoses of mental disorders according to DSM-IV criteria [35, 36].
- We will establish how long the current depressive episode has been evolving and any previous history of depression.

At the baseline and the follow-up interviews, we will also measure:

- The degree of disability caused by the psychological disorder will be evaluated using the Sheehan Disability Inventory (SDI) [37, 38]. This is a questionnaire with three items evaluating the disability in the areas of work, social life, and family. Two additional items evaluate the degree of stress and the perception of social support. The first four items are measured using visual analogue scales, from 0 (no effect) to 10 (extreme effect). Social support is evaluated in a scale from 0 % (non-existent social support) to 100 % (ideal social support).
- The patient’s perspective on the efficacy of treatment will be assessed using the self-report Patient Global Impression of Change (PGIC) [39]. This is a 7 point scale depicting a patient’s rating of overall improvement (from “very much improved” to “very much worse”).
- Patient satisfaction with the care will be evaluated using a single item: “How would you rate the quality of the care you have received (or how they have dealt with you) for your pain and/or depression problems?” with five response options in a Likert scale, from “excellent” to “bad” [40].
- Pharmacological treatments for pain and depression and use of health resources will be determined by means of interviewing the patient and exploring his/her computerised clinical records (i.e., prescription and pharmacy billing, number and type of primary care, specialist and emergency visits and hospitalisations for pain-related problems or depression-related problems).

### Statistical methods

#### Sample size

We will consider the response rates to treatment for depression and pain as reference variables. We will assume that the proportion of patients achieving a therapeutic response will be 50 % for both outcomes in the control group [14], and we expect a difference ≥17 % in the intervention group. Accepting an alpha risk of 0.05 and a beta risk of 0.2 in a two-tailed test, and with an expected loss of 15 %, 154 subjects are enough for each group. We will use the following formula to correct this figure for the design effect (randomisation by clusters): Deff = 1 + (m - 1) × ICC, where Deff: design effect; m: cluster size; and ICC: intraclass correlation coefficient [41]. Assuming an ICC of 0.01 [14] and m = 8, the Deff will be 1.07. As such, each study group will require 165 patients (1.07 × 154 = 165), distributed into 21 clusters of 8 patients each.

#### Analysis strategy

The randomisation will take place at the level of the primary care physicians (clusters), while the results of the intervention will be analysed at the individual-patient level [41]. The principal analyses will be by intention-to-treat, considering the patients from each group according to the initial assignment to the study groups, independently of compliance with the programme by either the individual patient or the physician.

We will compare the intervention group with the control group in order to verify that they are comparable in their baseline characteristics.

The main outcomes (dependent) variables are depressive symptoms (HSCL-20 score), response rate to the depression treatment, remission rate for depression, severity and interference of the pain (BPI), therapeutic response rate for the pain, and health-related quality of life at 3, 6, and 12 months.

In order to evaluate the effects of the intervention on the dichotomous variables we will use multilevel logistical regression models with mixed effects, adjusted by cluster, and employing an estimation of the Odds Ratio (95 % CI) of the intervention group with respect to the control group as a measurement of the effect. In order to measure the effect on the continuous variables we will use linear regression models with random effects (cluster), estimating the differences in adjusted averages (95 % CI) for the intervention group versus control group. For the main outcome variables we will calculate the intracluster correlation coefficient (ICC). Statistical significance will be established as *p <* 0.05. We will use STATA-12 and SPSS v.15 software for these calculations. This analysis strategy takes into account the design effect (randomisation by clusters) for any potential lack of independence among observations within the same primary care centre.

## Trial Status and forecast execution dates

Initial recruitment of patients: June 2015; deadline for recruitment of patients: December 2016; deadline for period of patient monitoring: December 2017; publication of the results: January-June 2018. The study is ongoing and currently we are recruiting patients (15 February, 2016; N^current^ = 130, out of a foreseen total sample of *n =* 330).

## Ethical aspects

The Study Protocol was approved by the Research Ethics Committee of the Jordi Gol i Gurina Primary Care Research Institute (IDIAP), Barcelona, on December 17^th^, 2014 (ref:P14/142).

The study design involves the need to obtain informed consent at two levels: a) from the collaborating primary care physicians; and b) from the participating patients.

The information provided by the research team to the healthcare professionals will include the voluntary nature of participation and the ability to leave the study without any negative consequences for the health care of their patients. The participating professionals will sign a document stating their commitment to keep patients informed, verifying the absence of conflicts of interest, and specifying that their participation in the study will promote the interests of their patients.

The written information provided by the study interviewer to the patients prior to their consent will include a general overview of the study’s objectives and activities; the voluntary nature of his or her participation; the right to leave the study at any time with a guarantee that they will continue to receive the treatment their physician considers most appropriate; and the guarantee that patients included in the control arm will receive the treatment that their physician considers to be the most appropriate, with no restrictions.

If the patient withdraws consent to receive periodic study assessment, they will be considered to have dropped out and will not be contacted for periodic assessment in the future.

All data will be securely held with restricted access and clinical information will not be used beyond the objectives of the study.

## Discussion

A study performed in the United States has demonstrated that a programme for the integrated care of depression and chronic pain can produce improved health-related results [18]. However, we are unaware of any subsequent studies that have replicated these results in other environments, particularly within European healthcare systems. The objective of this project is to develop a programme for managing depression and chronic musculoskeletal pain that is feasible and applicable in the primary care setting within the Spanish healthcare system, and to evaluate its clinical effectiveness. These objectives fit particularly well with the strategies currently being promoted by Spanish and European healthcare authorities [42, 43] to strengthen and improve the approach to chronic conditions, and specifically for patients with chronic conditions involving comorbidity and complexity.

This project raises some methodology issues that should be commented upon. Firstly, randomised assignment by clusters has been chosen [41] because the intervention to be evaluated is largely designed to be applied at the professional level (e.g., support from the care manager in the effective use of the computerised clinical guide for depression), while the results of the intervention are measured in each patient in the form of health-related outcomes. This design seeks to avoid any decrease in the effects of the intervention that could be caused by possible contamination among the study groups if the randomisation were to take place on an individual basis, in which case the same physician would have to provide care to patients assigned to both the intervention and control groups.

Secondly, the procedure used to identify and recruit patients eligible for the study is based on a screening. This prevents any possible selection bias that could exist if the participating physicians themselves recruited the patients (e.g., a physician from the intervention group might be more inclined that a physician from the control group to recruit patients who are more willing to comply with the treatment). The drawback is that this way of identifying patients does not correspond with the manner to diagnose chronic pain and depression established in usual practice, and this could have a certain effect on the representativeness of the sample. Furthermore, the fact that individuals are recruited at a point in time when they are not actively seeking medical care could cause the sample to include patients who are not strongly motivated when it comes to the intervention, particularly towards the psychoeducational programme which requires a certain level of commitment and personal effort by the patient.

Thirdly, the intervention incorporates several components. It will not be possible to determine which one is responsible of any observed change/improvement, or even whether the results are due to the non-specific effects.

The designed intervention relies upon a structured package of multiple strategies: structured and optimised management of depression following clinical guides, the care management, and a psychoeducational intervention used to provide patients with the resources and skills to self-manage their pain and depressive symptomatology. One of the most innovative aspects is that of defining and promoting the role of the care manager in the systematic, structured management of the chronic health problems experienced by our patients. The innovations incorporated into this intervention take into account its feasibility under the typical primary care conditions found in Spain, so that if the results of the evaluation are favourable, generalised implementation of the programme will be feasible.

## Ethics approval and consent to participate

Study Protocol approved by the Research Ethics Committee of the Jordi Gol i Gurina Primary Care Research Institute (IDIAP), Barcelona, on December 17^th^, 2014 (ref:P14/142). The study design involves written informed consent from the collaborating primary care physicians and from the participating patients.

## Open access

This article is distributed under the terms of the Creative Commons Attribution 4.0 International License (http://creativecommons.org/licenses/by/4.0/), which permits unrestricted use, distribution, and reproduction in any medium, provided you give appropriate credit to the original author(s) and the source, provide a link to the Creative Commons license, and indicate if changes were made. The Creative Commons Public Domain Dedication waiver (http://creativecommons.org/publicdomain/zero/1.0/) applies to the data made available in this article, unless otherwise stated.

## Abbreviations

BPI: brief pain inventory
Deff: design effect
DSM-IV: diagnostic and statistical manual of mental disorders, fourth edition
DUSOI: duke severity of illness checklist
eCAP: Primary care clinical station (in Catalan: Estació clínica d’atenció primària, i. E., the computerized platform of primary care clinical records)
EuroQol-5D: European quality of life, 5 dimensions
GAF: global assessment of functioning scale
HSCL-20: hopkins symptom checklist, 20 items
ICC: intraclass coefficient correlation
ICD-10: international classification of diseases, 10th edition
IDIAP: primary care research institute (in catalan: institut d’investigació en atenció primària)
MINI: mini international neuropsychiatric interview
PGIC: patient global impression of change
PHQ-9: patient health questionnaire, 9 items
PRIME-MD: primary care evaluation of mental disorders
SDS: sheehan disability scale

## References

[1] Fernández A, Saameño JA, Pinto-Meza A, Luciano JV, Autonell J, Palao D (2010). Burden of chronic physical conditions and mental disorders in primary care. Br J Psychiatry.

[2] Serrano-Blanco A, Palao DJ, Luciano JV, Pinto-Meza A, Luján L, Fernández A (2010). Prevalence of mental disorders in primary care: results from the diagnosis and treatment of mental disorders in primary care study (DASMAP). Soc Psychiatry Psychiatr Epidemiol.

[3] Calsina-Berna A, Moreno Millán N, González-Barboteo J, Solsona Díaz L, Porta SJ (2011). Prevalencia de dolor como motivo de consulta y su influencia en el sueño: experiencia en un centro de atención primaria. Aten Primaria.

[4] Hasselström J, Liu-Palmgren J, Rasjö-Wrååk G (2002). Prevalence of pain in general practice. Eur J Pain.

[5] Miró J, Paredes S, Rull M, Queral R, Miralles R, Nieto R (2007). Pain in older dults: a prevalence study in the Mediterranean region of Catalonia. Eur J Pain.

[6] Aragonès E, Piñol JL, Labad A, Masdéu RM, Pino M, Cervera J (2004). Prevalence and determinants of depressive disorders in primary care practice in Spain. Int J Psychiatry Med.

[7] Aragonès E, Labad A, Piñol JL, Lucena C, Alonso Y (2005). Somatized depression in primary care attenders. J Psychosom Res.

[8] Caballero L, Aragonès E, García-Campayo J, Rodríguez-Artalejo F, Ayuso-Mateos JL, Polavieja P (2008). Prevalence, characteristics, and attribution of somatic symptoms in Spanish patients with major depressive disorder seeking primary health care. Psychosomatics.

[9] Bair MJ, Robinson RL, Katon W, Kroenke K (2003). Depression and pain comorbidity: a literature review. Arch Intern Med.

[10] Kroenke K, Wu J, Bair MJ, Krebs EE, Damush TM, Tu W (2011). Reciprocal relationship between pain and depression: a 12-month longitudinal analysis in primary care. J Pain.

[11] Urquhart DM, Hoving JL, Assendelft WW, Roland M, van Tulder MW (2008). Antidepressants for non-specific low back pain. Cochrane Database Syst Rev.

[12] Coventry PA, Hudson JL, Kontopantelis E, Archer J, Richards DA, Gilbody S (2014). Characteristics of effective collaborative care for treatment of depression: a systematic review and meta-regression of 74 randomised controlled trials. PLoS One.

[13] Sighinolfi C, Nespeca C, Menchetti M, Levantesi P, Belvederi Murri M, Berardi D (2014). Collaborative care for depression in European countries: a systematic review and meta-analysis. J Psychosom Res.

[14] Aragonès E, Piñol JL, Caballero A, López-Cortacans G, Casaus P, Hernández JM (2012). Effectiveness of a multi-component programme for managing depression in primary care: a cluster randomized trial. The INDI project. J Affect Disord.

[15] Lamb SE, Hansen Z, Lall R, Castelnuovo E, Withers EJ, Nichols V (2010). Group cognitive behavioural treatment for low-back pain in primary care: a randomised controlled trial and cost-effectiveness analysis. Lancet.

[16] Turk DC, Swanson KS, Tunks ER (2008). Psychological approaches in the treatment of chronic pain patients—when pills, scalpels, and needles are not enough. Can J Psychiatry.

[17] Warsi A, LaValley MP, Wang PS, Avorn J, Solomon DH (2003). Arthritis self-management education programs: a meta-analysis of the effect on pain and disability. Arthritis Rheum.

[18] Kroenke K, Bair MJ, Damush TM, Wu J, Hoke S, Sutherland J, et al. Optimized antidepressant therapy and pain self-management in primary care patients with depression and musculoskeletal pain: a randomized controlled trial. JAMA. 2009;301:2099–110. 10.1001/jama.2009.723 PMC2884224 19470987

[19] Rothman AA, Wagner EH (2003). Chronic Illness Management: What Is the Role of Primary Care?. Ann Intern Med.

[20] Adaptació al model sanitari català de la guia de pràctica clínica sobre el maneig de la depressió major en l’adult [Adaptation to the Catalan health model of clinical practice guideline for the management of major depression in adults]. Barcelona: AIAQS & Pla director de salut mental i addiccions. Dept de Salut. Generalitat de Catalunya; 2010. Available at: http://aquas.gencat.cat/web/.content/minisite/aquas/publicacions/2010/pdf/adaptacio_gpc_depressio_aiaqs_2010ca.pdf. Last Accessed: 10 March 2016.

[21] Grupo de Trabajo sobre el Manejo de la Depresión Mayor en el Adulto. Guía de Práctica Clínica sobre el Manejo de la Depresión Mayor en el Adulto [Clinical Practice Guideline on the Management of Major Depression in Adults]. Madrid: Plan Nacional para el SNS del MSC. Avalia-t; 2008. Guías de Práctica Clínica en el SNS: avalia-t N° 2006/06

[22] ECAP Central blog. Nova Guia de la Depressió Major a l’ECAP. Resum Guia de la Depressió a l’ECAP. [New Guideline about Depression in ECAP. Abstract of the Guideline on Depression in ECAP] April 2014. Available at: https://ecapcentral.wordpress.com/tag/gpc/. Last Accessed: 10 March 2016.

[23] Gatchel RJ (2005). Clinical Essentials of Pain Management.

[24] Turk DC, Gatchel RJ (2002). Psychological approaches to pain management. A practitioner’s handbook (2nd edition).

[25] Williams JW, Stellato CP, Cornell J, Barrett JE (2004). The 13- and 20-item Hopkins Symptom Checklist Depression Scale: psychometric properties in primary care patients with minor depression or dysthymia. Int J Psychiatry Med..

[26] Keller MB (2003). Past, present, and future directions for defining optimal treatment outcome in depression: remission and beyond. JAMA.

[27] Unutzer J, Katon W, Callahan CM, Williams JW, Hunkeler E, Harpole L (2002). Collaborative care management of late-life depression in the primary care setting: a randomized controlled trial. JAMA.

[28] Cleeland C (2009). The Brief Pain Inventory: user guide.

[29] Badia X, Muriel C, Gracia A, Núñez-Olarte JM, Perulero N, Gálvez R (2003). Validation of the Spanish version of the Brief Pain Inventory in patients with oncological pain. Med Clin (Barc).

[30] Badia X, Roset M, Montserrat S, Herdman M, Segura A (1999). La versión española del EuroQol: descripción y aplicaciones. Med Clin (Barc).

[31] Cabasés JM (2015). The EQ-5D as a measure of health outcomes. Gac Sanit.

[32] Domingo-Salvany A, Regidor E, Alonso J, Alvarez-Dardet C (2000). Una propuesta de medida de la clase social. Aten Primaria.

[33] Parkerson GR, Broadhead WE, Tse CK (1993). The Duke Severity of Illness Checklist (DUSOI) for measurement of severity and comorbidity. J Clin Epidemiol.

[34] Martínez C, Juncosa S, Roset M (1998). ¿Está relacionada la gravedad con la utilización de recursos? Una exploración del Duke Severity of Illness Scale (DUSOI). Aten Primaria.

[35] Spitzer RL, Williams JB, Kroenke K, Linzer M (1994). deGruy FV 3rd, Hahn SR, et al. Utility of a new procedure for diagnosing mental disorders in primary care. The PRIME-MD 1000 study. JAMA.

[36] Baca E, Saiz J, Aguera L, Caballero L, Fernandez-Liria A, Ramos J (1999). Validation of the Spanish version of PRIME-MD: a procedure for diagnosing mental disorders in primary care. Actas Esp Psiquiatr.

[37] Sheehan DV, Harnett-Sheehan K, Raj BA (1996). The measurement of disability. Int Clin Psychopharmacol.

[38] Luciano JV, Bertsch J, Salvador-Carulla L, Tomás JM, Fernández A, Pinto-Meza A (2010). Factor Structure, Internal Consistency and Construct Validity of the Sheehan Disability Scale in a Spanish Primary Care Sample. J Eval Clin Pract.

[39] Dworkin RH, Turk DC, Wyrwich KW, Beaton D, Cleeland CS, Farrar JT (2008). Interpreting the clinical importance of treatment outcomes in chronic pain clinical trials: IMMPACT recommendations. J Pain.

[40] Ware JE, Hays RD (1988). Methods for measuring patient satisfaction with specific medical encounters. Med Care.

[41] Ukoumunne OC, Gulliford MC, Chinn S, Sterne JAC, Burney PGJ (1999). Methods for evaluating area-wide and organisation-based interventions in health and health care: a systematic review. Health Technol Assess.

[42] Ferrer Arnedo C, Orozco Beltrán D (2012). Román Sánchez P (coord.) Estrategia para el Abordaje de la Cronicidad en el Sistema Nacional de Salud.

[43] Busse R, Blümel M, Scheller-Kreinsen D, Zentner A. Tackling chronic disease in Europe. Strategies, interventions and challenges. European Observatory on Health Systems and Policies. Observatory Studies Series, N° 20. Copenhagen: WHO Regional Office for Europe; 2010.

